# Comparison of the Analgesic Efficacy of Ultrasound-Guided Quadratus Lumborum Block and Ilioinguinal-Iliohypogastric Nerve Block in Paediatric Patients After Inguinal Hernia Surgery: A Prospective Randomized Controlled Trial

**DOI:** 10.4274/TJAR.2023.231289

**Published:** 2023-10-24

**Authors:** Mustafa Altınay, Hacer Şebnem Türk

**Affiliations:** 1 University of Health Sciences Turkey, Şişli Hamidiye Etfal Training and Research Hospital, İstanbul, Turkey

**Keywords:** Inguinal hernia, nerve block, paediatric anaesthesia, pain, regional anaesthesia

## Abstract

**Objective:**

To compare the postoperative analgesic efficacy of quadratus lumborum block (QLB) and ilioinguinal-iliohypogastric nerve block (IIIHB) in paediatric patients who have undergone unilateral inguinal hernia surgery.

**Methods:**

This prospective randomized controlled study was designed in a single center and included 60 paediatric patients aged 2-7 years who had undergone inguinal hernia repair surgery and received an American Society of Anesthesiologists score of 1-2. Patients were randomized into two groups: those receiving ultrasound-guided QLB and those receiving IIIHB. The primary outcomes of the study were patients’ face, legs, activity, cry, and consolability (FLACC) scores at 1, 2, 6, 12, and 24 hours post-surgery.

**Results:**

The mean heart rate 15 and 30 minutes post-surgery in the QLB group was lower than that of the IIIHB group, and the difference at both times was statistically significant (*P* < 0.001). The mean FLACC score of the QLB group was lower than that of the IIIHB group at 6, 12, and 24 hours post-surgery, and the differences were statistically significant (*P*=0.004, *P*=0.006, and *P* < 0.001, respectively). Between the groups, there was no statistically significant difference in the number of patients who were administered rescue analgesics or oral ibuprofen, the time of first ibuprofen administration, or the frequency of complications (*P*=1.000, *P*=0.145, *P*=0.195, and *P*=1.000, respectively).

**Conclusion:**

Compared with IIIHB, QLB achieves superior postoperative analgesic effects in paediatric patients who have undergone inguinal hernia surgery, as evidenced by longer analgesic periods, lower pain scores, and lower analgesic consumption.

**Main Points**

• Quadratus lumborum block (QLB) achieves longer analgesic duration, lower pain scores, and lower analgesic consumption than ilioinguinal-iliohypogastric nerve block (IIIHB) in paediatric patients who have undergone inguinal hernia surgery.

• Both QLB and IIIHB are effective and safe block methods.

• A significant decrease in heart rate at 15 and 30 minutes post-surgery in patients receiving QLB compared with IIIHB may be associated with the former’s greater analgesic effect.

## Introduction

Perioperative and postoperative pain management has often been neglected in paediatric patients.^1^ Effective perioperative and postoperative analgesic techniques reduce surgical stress and contribute to a physiologically and psychologically more comfortable postoperative experience, shorter recovery and hospitalization times, and positive long-term personality development in children.

Children’s physiological, pharmacokinetic, and pharmacodynamic differences from adults delay the metabolization and elimination of systemic analgesics, which can result in prolonged analgesic effects and an increased risk of apnea.^2^ Regional anaesthesia and central block procedures minimize systemic opioid consumption, reduce adverse drug events, and ensure efficient and safe analgesia; however, some central block procedures (e.g., caudal block) have disadvantages, including motor block and urinary retention.^3^

Inguinal hernia surgery is one of the most common paediatric surgical procedures. Peripheral block techniques, such as transversus abdominis plane (TAP) block and ilioinguinal-iliohypogastric nerve block (IIIHB), have been proven to safely and effectively manage perioperative and postoperative pain in paediatric patients and have replaced central block techniques.^4,5^ In recent years, quadratus lumborum block (QLB) has emerged as a regional anaesthetic technique and been proven to promote analgesia in children as effectively as the caudal block procedure and more effectively than the TAP block.^6^ Nevertheless, the literature contains only one study comparing the effectiveness of IIIHB and QLB in paediatric patients.^7^

This study aims to compare the postoperative analgesic efficacies of QLB and IIIHB in paediatric patients who have undergone unilateral inguinal hernia surgery.

## Materials and Methods

Approval for this prospective randomized controlled study was obtained from the University of Health Sciences Turkey, Şişli Hamidiye Etfal Training and Research Hospital Ethics Committee (approval no: 3445, date: March 08, 2022) and from the Turkish Medicines and Medical Devices Agency on October 13, 2022 (approval no: 22-AKD-177). Clinical trials were initiated on October 28, 2022 upon registration with NCT05610943. Written and verbal consent was obtained from the parents of the children. All procedures were carried out in accordance with the ethical standards of the Declaration of Helsinki (2008), and the study was conducted according to Consolidated Standards of Reporting Trials guidelines.

### Inclusion Criteria

The study included 60 patients aged 2-7 years who received American Society of Anesthesiologists (ASA) physical scores of 1-2 and who were scheduled to undergo unilateral inguinal hernia surgery. Patients with coagulopathy, skin infection at the surgical site, a bupivacaine allergy, or a neuropsychiatric disease were excluded from the study.

### Sample Size and Randomization

Considering previous studies, the effect size [calculated as 1 according to the face, legs, activity, cry, and consolability (FLACC) score] taken as one unit of difference between the two groups after 24 hours (2 vs. 1) and the standard deviation of each group as 1, and the two-tailed t-test was calculated at a significance level of 0.05 and 95% power among independent groups and was determined to be 27 patients per group and 54 patients in total.^8^ A total of 60 patients were included, considering the margin of error.

Patients were randomized into two groups by the closed-envelope method using opaque envelopes prepared and sealed by study clinicians. The intervention group each patient would join was determined in the following manner: as patients were brought to the operating room, an envelope was selected in the preoperative admission area by a nurse blind to the entire study. All blocks were performed by the same anaesthesiologist. Patients’ FLACC scores were evaluated by a different anaesthesiologist who was blind to the blocks.

### Procedure

Premedication (midazolam) was administered orally at 0.5 mg kg^-1^ 30 minutes before surgery. Patients were then taken to the operating room, and their noninvasive arterial blood pressure, end-tidal carbon dioxide (CO_2_), peripheral oxygen saturation, and electrocardiogram readings were monitored continuously. Intravenous vascular access was opened with a 22-24-gauge cannula. After preoxygenation with a face mask, propofol (3 mg kg^-1^) and fentanyl (1 µg kg^-1^) were administered to induce anaesthesia. A ProSeal laryngeal mask of appropriate size based on each patient’s weight was deployed. Anaesthesia was maintained with a mixture of sevoflurane (2%), oxygen (50%), and air (50%).

The patients undergoing the QLB procedure was referred to as Group QLB, and the patients undergoing IIIHB were referred to as Group IIIHB. Block procedures were performed using an Esaote MyLab Five ultrasonography (USG) device (Florence, Italy) with a multifrequency linear probe (6-19 MHz) and a 22 g and 50 mm peripheral nerve block needle (Braun Sonoplex, Melsungen, Germany).

### Group QLB (n = 30)

A Type 2 (posterior) QLB was applied to patients in Group QLB. The patients were placed in a lateral position, with the side to be blocked in the superior position. To prevent airway complications during block application (e.g. when the patient’s position changed), an anaesthesiologist or senior assistant continuously monitored the patient’s head and laryngeal mask position. A sterile cover was placed at the injection site after skin antisepsis was ensured with 5% povidone-iodine. The USG probe was covered with a sterile sheath, and the probe was placed between the iliac crest and the costal margin. The external-internal oblique and transversus abdominis muscles were screened, and the probe was advanced through the posterior. The quadratus lumborum, psoas major, and erector spinae muscles were screened. The needle was advanced to the middle thoracolumbar fascia between the quadratus lumborum muscle and the erector spinae muscle by the in-plane technique, and the needle’s location was confirmed by injecting 1 mL of 0.9% saline solution. Following negative aspiration, 0.25% bupivacaine was injected at a dose of 0.5 mL kg^-1^.

### Group IIIHB (n = 30)

Patients in Group IIIHB were placed in a supine position. A sterile cover was placed after skin antisepsis was ensured with 5% povidone-iodine. The USG probe was covered with a sterile sheath and placed on the anterior abdominal wall parallel to the imaginary line between the umbilicus and the anterior superior iliac crest. After screening the external-internal oblique and transversus abdominis muscles, the IIIHB was screened as two small hypoechoic areas between the internal oblique muscle and the transversus abdominis muscle. The needle’s location was confirmed by injecting 1 mL 0.9% saline solution and advancing the needle toward the nerve structures using the in-plane technique. Following negative aspiration, 0.25% bupivacaine was injected at a dose of 0.2 mL kg^-1^.

### Postoperative Pain Management and Data Collection

Demographic data such as age, gender, and weight were recorded for all patients. Heart rate (HR) was recorded in both groups 15 and 30 minutes after block application. The time from induction of anaesthesia to awakening was recorded as the anaesthesia period; the time from cessation of sevoflurane administration to patient recovery was recorded as the recovery period; and the time from surgical incision to the final suture was recorded as the surgery period.

Data on the block technique used was collected by an anaesthesiologist blind to block procedure. Pain assessment was performed and FLACC scores were recorded 1, 2, 6, 12, and 24 hours after surgery, and patients were followed up for two hours in the recovery room. Fifteen mg kg^-1^ paracetamol rescue analgesia was administered intravenously to patients with FLACC scores ≥4 in the first two hours following surgery, and this was recorded. Two hours after surgery, patients were transferred to the ward, their oral intake was opened, and their FLACC scores were evaluated at 2^nd^, 6^th^, 12^th^, and 24^th^ hours post-surgery by an anaesthesiologist. Patients with FLACC scores ≥4 were administered 7 mg kg^-1^ of oral ibuprofen. The time of initial ibuprofen administration within the first 24 hours post-surgery was recorded. Complications such as nausea, vomiting, desaturation, bradycardia, tachycardia, hypotension, hematoma, and visceral damage from the procedure (e.g., intravascular puncture) were monitored and recorded.

### Primary Outcomes

The primary outcomes of the study were the FLACC scores of each group of patients 1, 2, 6, 12, and 24 hours post-surgery.

### Secondary Outcomes

The secondary outcomes of the study were perioperative HRs, the number of patients administered postoperative rescue analgesics, the number of patients administered postoperative oral ibuprofen, the time of the first postoperative administration of oral ibuprofen, and any complications that arose due to the block procedures used and/or postoperative use of systemic analgesics.

### Statistical Analysis

Statistical analysis was conducted using SPSS 15.0 (Armonk, New York, USA). The descriptive statistics captured were numbers and percentages (for categorical variables) and mean, standard deviation, minimum, maximum, and median (for numerical variables). The rates in the groups were compared with the chi-squared (χ^2^) test. Comparisons of numerical variables between the two groups were made with the Student’s t-test (when the normal distribution condition was met) and the Mann-Whitney U test (when the normal distribution condition was not met). Dependent group analyses were performed with repeated measurement analysis of variance (when the normal distribution condition was met) and the Friedman test (when the normal distribution condition was not met). Subgroup analyses were performed with the Wilcoxon test and interpreted with the Bonferroni correction. Alpha significance level was accepted at *P* < 0.05.

## Results

**Figure 1:**
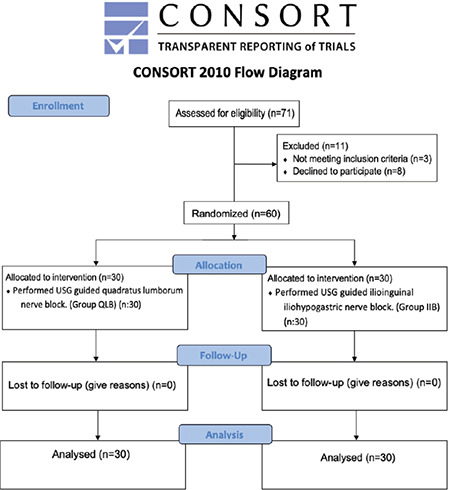
Consolidated Standards of Reporting Trials diagram comparing the postoperative analgesic efficacy of USG-guided quadratus lumborum block (QLB) and ilioinguinal-iliohypogastric nerve block (IIIHB) in infants undergoing inguinal hernia surgery. USG, ultrasonography.

Inguinal hernia surgery was performed on a total of 71 patients aged 2-7 years between November 2022 and March 2023. One patient was not included in the study due to an ASA score >3, two patients were not included due to the presence of neuropsychiatric disease, and eight patients who did not provide consent were not included in the study (Figure 1). The study ultimately included 60 patients. There was no statistically significant difference in the demographic findings (*P* > 0.05; Table 1) or in the anaesthesia administration, surgical process, or recovery periods (*P* > 0.05; Table 1) between the two groups.

There was no statistically significant difference between the mean HR values of the two groups at the beginning or after anaesthetization (*P*=0.082 and *P*=0.428, respectively; Table 2). The mean HR values of Group QLB after 15 and 30 minutes were lower compared with those of Group IIIHB, and the differences were statistically significant (*P* < 0.001 for each time mark; Table 2).

The mean FLACC score of Group QLB was found to be lower than that of Group IIIHB at 6, 12, and 24 hours post-surgery, and these differences were statistically significant (*P*=0.004, *P*=0.006, and *P* < 0.001, respectively; Table 3).

There was no statistically significant difference between the groups in the number of patients administered rescue analgesics and oral ibuprofen, the time of first administration of oral ibuprofen, or the frequency of complications (*P*=1.000, *P*=0.145, *P*=0.195, and *P*=1.000, respectively; Table 4).

## Discussion

The authors of this prospective randomized controlled study found that, in paediatric patients undergoing inguinal hernia surgery, the use of QLB achieved longer analgesic duration, lower pain scores, and less analgesic consumption than the use of IIIHB.

There was less need for oral ibuprofen in the first 24 hours post-surgery in Group QLB (6.7%) than in Group IIIHB (23.3%). The need for rescue analgesics occurred in two patients in Group IIIHB and one patient in Group QLB in the first two hours after surgery. Samerchua et al.^9^ compared the use of posterior QLB and IIIHB in paediatric patients who had undergone inguinal hernia surgery and similarly found the consumption of analgesics to be lower in the QLB group (the need for rescue analgesics was observed in one patient in the QLB group, versus five patients in the IIIHB group). Priyadarshini et al.^7^ evaluated the efficacy of lateral QLB against TAP block and IIIHB in paediatric patients who had undergone inguinal hernia surgery, using paracetamol for postoperative analgesia, and found no difference in total paracetamol consumption between groups. The same study used tramadol in patients who experienced pain despite the administration of paracetamol. Finding that 55% of the TAP block group, 35% of the IIIHB group, and 15% of the QLB group required additional tramadol, Priyadarshini et al.^7^ concluded that, compared with TAP block or IIIHB, the use of QLB leads to a decrease in opioid consumption in children undergoing inguinal hernia surgery. Data on total analgesic agent dosage were not included in the aforementioned study.

In this study, a postoperative analgesic was first required 12 hours after surgery in Group QLB and 7.4 hours after surgery in Group IIIHB. Comparatively, the mean time of first analgesic need post-surgery was 8.4 hours in the QLB group and 4.8 hours in the IIIHB group in the study by Samerchua et al.^9^, and the analgesia period was shorter. Priyadarshini et al.^7^ found that first analgesic need emerged 6 hours, 8 hours, and 12 hours after surgery in the TAP block, IIIHB, and QLB groups, respectively, a finding that corroborates the present study. As in this study, posterior QLB was performed in the study by Samerchua et al.^9^, whereas lateral QLB was performed in the study by Priyadarshini et al.^7^.

The analgesic efficacy of QLB has been associated with the spread of local anaesthesia through the middle thoracolumbar fascia. Due to the complex structure of the thoracolumbar fascia, local anaesthesia administered at the L4 level spreads to the endothoracic fascia through the medial-lateral arcuate and aortic hiatus. Although magnetic resonance imaging and cadaver studies have yielded different results, it is generally accepted that posterior QLB administration affords dermatomal spread between T11 and L1 and ensures somatic and visceral analgesia through paravertebral distribution and involvement of the ventral rami of the spinal nerves.^10,11^ The extent of dermatomal spread and visceral analgesia may explain QLB’s greater efficacy as an analgesic compared with IIIHB.

In a meta-analysis of seven randomized controlled studies comparing QLB with different analgesic techniques in paediatric patients who had undergone lower abdominal surgery, pain scores 2, 4, and 12 hours after surgery were found to be lower in the QLB group. Based on limited data, QLB achieved more efficient postoperative analgesia in lower abdominal surgery in paediatric patients.^12^ Additionally, a new meta-analysis of 69 randomized controlled studies comparing different regional anaesthesia techniques in paediatric patients who had undergone inguinal surgery found that QLB had the longest analgesia period.^13^

In the present study, postoperative pain was assessed by FLACC score, and FLACC scores at 0, 6, 12, and 24 hours postoperation were lower in Group QLB than in Group IIIHB. The mean FLACC score was <4 in both groups, indicating that IIIHB, too, is an effective regional anaesthetic technique after inguinal hernia surgery. No difference was found between the pain scores of the groups in the studies by Samerchua et al.^9^ or Priyadarshini et al.^7^, a fact that may be attributable to the smaller sample sizes of these studies compared with that of this study. Edwards et al.^14^ compared the analgesic efficacy of transmuscular QLB and IIIHB in adult inguinal hernia surgery patients^9^ and found that pain scores during activity and at rest were similar 24 hours after surgery and that analgesic duration and the time of first opioid consumption post-surgery was similar in both groups, a result that deviates from the findings of paediatric studies. Edwards et al.^14^ used a similar dose and volume of local anaesthesia in both groups, and adjuvant clonidine was added.

With the increasing use of USG in regional anaesthesia application, the search for minimum dosages and volumes that are still effective and safe has emerged. In the present study, 0.25% of bupivacaine was administered at 0.5 mL kg^-1^ in Group QLB and 0.2 mL kg^-1^ in Group IIIHB. Unfortunately, there are insufficient data on the pharmacodynamic efficacy and systemic toxicity of local anaesthetic agents in children.^15^ Willschke et al.^16^ conducted a study to determine the optimal volume for IIIHH administration under ultrasound guidance in paediatric patients and showed that, for IIIHH, ultrasound-guided local anaesthetic volume could be reduced to 0.075 mL kg^-1^ in children. These different doses and volumes were selected according to studies on effective dose in paediatric patients.^15,16,17,18,19^ Samerchua et al.^9^ employed similar doses of bupivacaine. Priyadarshini et al.^7^, by contrast, administered 0.25% ropivacaine at 0.2 mL kg^-1^ in the IIIHB group and 0.4 mL kg^-1^ in the QLB group. Using similar dosages and volumes for both blocks, Edwards et al.^14^ found the analgesic efficacy of QLB and IIIHB to be similar in adult patients. Alternatively, Mostafa et al.^20^ used similar doses and volumes to compare the analgesic efficacy of QLB and IIIHB in children who had undergone inguinal hernia surgery and found lower postoperative pain scores and less analgesic consumption in the QLB group. Mostafa et al.^20^ attributed this outcome to the wide dermatomal dissemination and visceral-somatic analgesic activity of QLB.

In the present study, HRs at 15 and 30 minutes after block procedures were lower in Group QLB than in Group IIIHB. Both groups had a decrease in HR at all times compared with baseline and after anaesthesia. It is known that effective anaesthetic techniques provide perioperative hemodynamic control; thus, a greater decrease in HR at 15 and 30 minutes in Group QLB may be associated with greater analgesic effect. It has been suggested that local anaesthetic spread to the paravertebral area in QLB and dermatomal involvement in the wider area may be associated with hypotension.^21,22^ Arterial blood pressure monitoring data were not evaluated in this study.

No complications related to regional anaesthesia procedures were observed in this study. Postoperative vomiting was observed in two patients in Group IIIHB and one patient in Group QLB. Samerchua et al.^9^ observed vascular intervention in the IIIHB group in only one patient. No complications were observed in the study by Priyadarshini et al.^7^. In both studies, only procedure-associated complications were evaluated.

### Study Limitations

A limitation of this study is absence of block performance times, such that sensory block levels could not be evaluated and total analgesic consumption could not be recorded. Another limitation of the study is its exclusion of hemodynamic parameters such as blood pressure, end-tidal CO_2_, and oxygen saturation, as it is not sufficient to comment on hemodynamic changes based on HR alone.

## Conclusion

In paediatric patients undergoing inguinal hernia surgery, QLB provides longer analgesic duration, lower pain scores, and lower analgesic consumption than IIIHB. More randomized controlled studies and meta-analyses are needed to determine the greater analgesic effect of QLB in paediatric patients.

## Figures and Tables

**Table 1:**
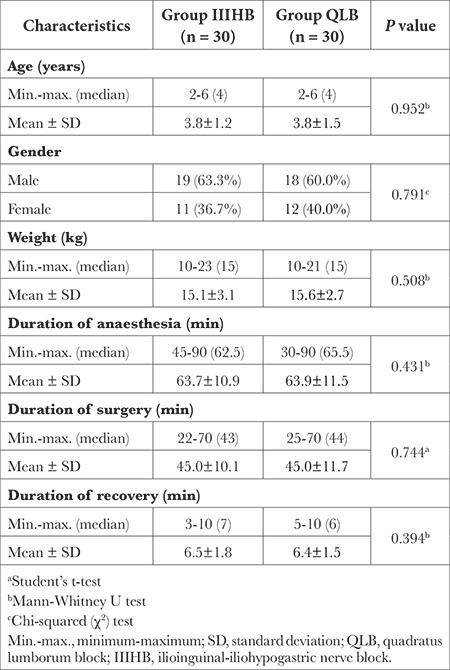
Demographic Data and Time Period Comparisons Between Group QLB and Group IIIHB

**Table 2:**
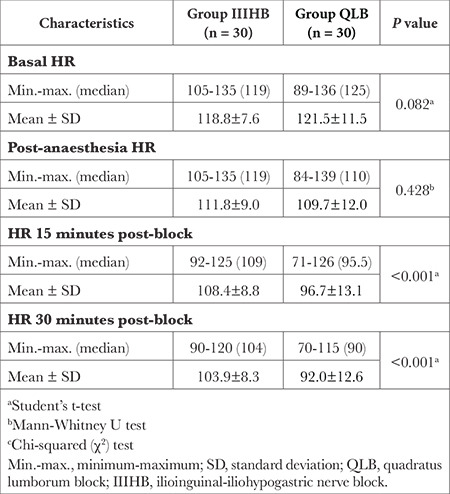
Comparison of Heart Rates (beats min^-1^) Between Group QLB and Group IIIHB

**Table 3:**
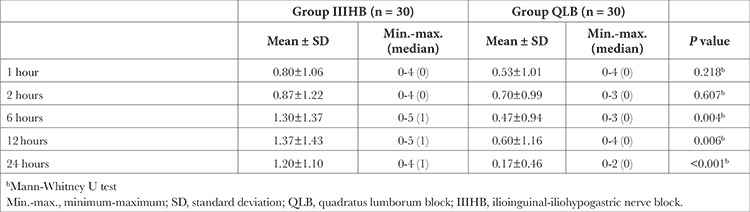
Comparison of Face, Legs, Activity, Cry, and Consolability Scores Between Group QLB and Group IIIHB

**Table 4:**
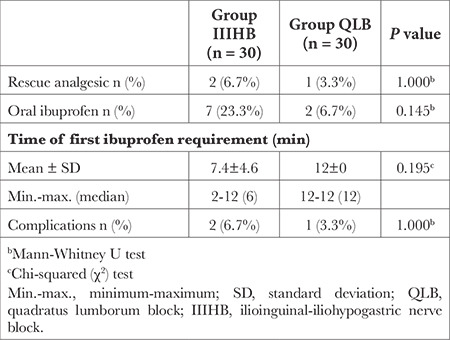
Comparison of Oral Ibuprofen Need, Time of First Analgesic Requirement, Rescue Analgesic Requirement, and Complications Between Group QLB and Group IIIHB
